# A Comparison Study of Nutritional Assessment, Diet and Physical Activity Habits, Lifestyle and Socio-Demographic Characteristics in Individuals with and without Dizziness/Vertigo

**DOI:** 10.3390/nu15184055

**Published:** 2023-09-19

**Authors:** Ayse Gunes-Bayir, Zelal Tandogan, Özge Gedik-Toker, Aysegul Yabaci-Tak, Agnes Dadak

**Affiliations:** 1 Department of Nutrition and Dietetics, Faculty of Health Sciences, Bezmialem Vakif University, Eyüpsultan, 34065 Istanbul, Turkey; 2 Division of Nutrition, Institute of Health Sciences, Istanbul University, Fatih, 34093 Istanbul, Turkey; zelal.tandogan@ogr.iu.edu.tr; 3 Department of Audiology, Faculty of Health Sciences, Bezmialem Vakif University, Eyüpsultan, 34065 Istanbul, Turkey; ogediktoker@bezmialem.edu.tr; 4 Department of Biostatistics, Faculty of Medicine, Bezmialem Vakif University, Fatih, 34093 Istanbul, Turkey; ayabaci@bezmialem.edu.tr; 5 Institute of Pharmacology and Toxicology, University of Veterinary Medicine Vienna, 1210 Vienna, Austria; agnes.dadak@vetmeduni.ac.at

**Keywords:** dizziness, vertigo, obesity, dietary habits, physical activity habits, lifestyle, nutritional assessment

## Abstract

Dizziness and vertigo are growing health problems and have become responsible for increases in health expenditures. In this context, a case-control study has been conducted by nutritional assessment, including dietary and physical activity habits, lifestyle, and socio-demographic characteristics in adults with (patient group) and without (control group) dizziness or vertigo, and the outcomes were compared between these groups. The patient (*n* = 150) and control (*n* = 150) groups included 300 participants. The 24-h Dietary Recall and the food frequency questionnaire (FFQ-21) were conducted in order to gain detailed information about foods and beverages consumed by the participants. Additionally, a questionnaire was completed, assessing general socio-demographic (age, gender, etc.) and lifestyle (smoking, alcohol consumption, and obesity) characteristics, anthropometric measurements, and dietary and physical activity habits. The results revealed that there is an association between dizziness/vertigo and female gender and increasing age. Smoking status and alcohol consumption did not differ between the groups, whereas differences in body mass index and obesity were significantly higher in the patient group (65%; *n* = 98) than the control group (46%; *n =* 69) (*p =* 0.001). Skipping meals “everyday” was significantly high (*p =* 0.044), and lunch was the most skipped meal in the patient group. The three most preferred cooking methods were oven baking, boiling, and frying for both groups. Daily water intake in the patient group was lower than in the control group (*p =* 0.026). Dietary intake for carotene and vitamin K were significantly lower in the patient group than the control group, but the opposite was true for vitamin D intake (*p <* 0.05). Daily consumption of bread and dairy products were highest in the patient group (*p <* 0.05). The physical activity rate was 35% (*n =* 53) in the control group and 28% (*n =* 42) in the patient group. Regular walking was the most preferred activity in both groups (*p =* 0.037). Active monitoring of individual diet and hydration along with supporting professional counseling are advisable. In addition, a healthy lifestyle including weight control and regular physical activity can be helpful to reduce symptoms of dizziness/vertigo.

## 1. Introduction

A number of studies have shown that vertigo (including vertigo and non-vestibular vertigo) is among the most common complaints in medicine, affecting approximately 20–30% of the general population and about two to three times more women than men [1]. Dizziness is a general term for a balance disorder often associated with lightheadedness. A specific subtype of dizziness that creates the false sense that the surroundings are spinning or moving is called vertigo. Vertigo has a considerable impact on individuals and can be a symptom of a variety of conditions with different etiologies [2,3]. Vestibular vertigo was defined as rotational vertigo (illusion of self-motion or object motion), positional vertigo (vertigo or dizziness provoked by changes of head position, such as lying down or turning in bed), or recurrent dizziness with nausea and oscillopsia or imbalance [2]. The potential benefit of studies investigating general risk factors for dizziness/vertigo is limited, and findings must be interpreted cautiously. Nevertheless, some interesting insights can result from such studies. The prevalence of dizziness is 14–30% in Turkey, according to a large survey-based study [4]. Although vestibular disorders commonly increase with age [5], approximately 5% of children and adolescents complain about dizziness/vertigo and balance problems [6,7]. Symptoms of vertigo can occur with varying frequencies, from only once over a certain time period (monophasic) with recurrent attacks (episodic) to persistently [7,8], whereas patients with dizziness have difficulty describing the quality of their symptoms but can more consistently identify the timing and triggers [8].

The general cause of vertigo in individuals is positional vertigo (PV), and its most frequent type is benign paroxysmal positional vertigo (BPPV), which is more widespread in older individuals than in other age groups [9]. However, vertigo can also be an indicator of Meniere’s disease (MD), which is characterized by spontaneous recurrent vertigo, fluctuating sensorineural hearing loss, aural fullness, and tinnitus [10]. Vertigo is often the chief symptom of MD, including nausea, vomiting, and sweating [11]. As a result, the management of MD aims to reduce the incidence and severity of vertigo. MD accounts for 3% of those admitted to the hospital with dizziness [1].

It has been emphasized in studies that vertigo is an emerging health concern, especially affecting healthy aging, and is thus responsible for a gradual increase in healthcare utilization [3,8,10,11]. Dizziness/vertigo not only impairs patient functioning in daily life by increasing their risk of falling [12] but is also frequently associated with depression and anxiety [1]. Vertigo is a common but inconclusive symptom, and the patient’s anamnesis is important in its diagnosis [8]. Since differential diagnosis is broad, thorough physical examination is essential. Additional laboratory testing and imaging can also be helpful for the evaluation of patients with dizziness. For accurate diagnosis, it may sometimes be necessary to monitor the patient to capture acute phases.

A cohort study conducted over the period 2002–2019 investigated the relationship between some lifestyle characteristics such as smoking, alcohol consumption, and obesity in a MD ≥ 40 year–old cohort in Korea [13]. Results showed that smoking was positively associated with MD, while alcohol consumption was negatively related to an increased risk of MD. On the other hand, there was no association between obesity and MD in the same study. In contrast, another study comparing cross-sectional data from the UK Biobank of MD patients and non-MD patients suggested that the obesity rate was higher in MD patients (33%) than in non-MD individuals (27.6%) [14].

A number of studies suggested that nutritional imbalance may be related to dizziness/vertigo [15,16,17]. A study conducted in Brazil has found a relationship between BPPV and a diet rich in carbohydrates and polyunsaturated fatty acids, along with insufficient dietary fiber intake, in the elderly [16]. Approximately 31% of BPPV patients had also inadequate feeding. Decreased serum vitamin D may be a risk factor of BPPV [15]. Recently, a study has reported that individuals aged 40 years and older suffering from vertigo showed significantly lower intake for carotene, vitamin A, and vitamin B2, whereas dietary fiber intake of these patients was not significantly different in comparison to non-vertigo individuals in Korea [17]. A meta-analysis revealed that an intake of 400 IU vitamin D and 500 mg calcium carbonate twice a day for 1 year significantly resulted in the recurrences of vertigo in 24% of BPPV patients [9]. 

A significant association was found between the lack of regular physical activity and dizziness/vertigo [18,19]. Interestingly, a study on BPPV showed no significant association between lack of physical activity and BPPV in men, but a statistically significant association in women [16]. In general, regular physical activity can greatly contribute to improvement of quality of life [20].

Based on the considerations presented, an investigation of the nutritional assessment, including diet, lifestyle, and physical activity habits, in adults with dizziness/vertigo (patient group) and without dizziness/vertigo (control group) was the first aim of the present study, and the outcomes were compared between these groups as the second aim of this study. In this context, intakes for energy, macronutrients, and micronutrients by nutritional assessment; dietary habits, including skipping meals; the consumption of salt and water; the most preferred food groups; the most preferred cooking methods; the absence or presence of physical activity habits (consisting of type, frequency, and duration of physical activity); and lifestyle factors, such as obesity, alcohol consumption, and smoking of both groups were evaluated and compared. Additionally, socio-demographic data of study participants were also examined and compared between the groups.

The data collected in this study will help shed light on health strategies that can help reduce negative consequences of symptom management and promote beneficial effects in individuals with dizziness or vertigo.

## 2. Materials and Methods

### 2.1. Study Population

Taking the previous studies as a reference [15,16], the sample size was calculated as *n*1 = *n*2 = 130 and *n* = 260 individuals for the 1.6-unit difference between the means with standard deviations taken as 3.4 and 3.6, respectively, for 80% power at 95% confidence level and 0.05 significance level. A total of 300 individuals participated in this case-control study; 150 patients suffering from vertigo were allocated to the patient group and 150 individuals without dizziness/vertigo to the control group. The study population was applicant patients and patient companions in the Bezmialem Vakif University Hospital (Istanbul, Turkey) in 2017–2021. 

Adults between the ages of 18 and 55, without any diagnosis of systemic, neurological, endocrine, or cardiovascular disease, and who applied to audiology outpatient clinics with complaints of dizziness/vertigo, were determined as the patient group of this study. Necessary vestibular evaluations were made after anamnesis. They were asked questions to help with diagnosis, such as whether he/she experienced a sensation that the environment around them was spinning, how he/she described his/her vertigo, whether he/she had an attack, and, if so, how long the attacks lasted, how the attacks started, and whether there was accompanying hearing loss [21]. Patients who were not diagnosed with any systemic, neurological, endocrine, or cardiovascular diseases were included in this study. However, since the aim of the study was to questions the lifestyle and some habits of individuals with dizziness/vertigo regardless of the underlying mechanism, adults with this complaint were not classified according to pathology.

Individuals aged between 18 and 55 years without complaints of dizziness/vertigo and who were not diagnosed with any systemic, neurological, endocrine, or cardiovascular diseases were included in the control group of the present study. Prior to enrolment, the purpose of the research and the necessary information were given to the participants, and written informed consent was obtained from the participants. 

The exclusion criteria for both groups in this study were individuals not aged between 18 and 55 years, pregnant, having communication difficulties, and who have had ear surgery.

### 2.2. Data Collection

The survey used in this study was modified from the studies of Gunes-Bayir et al. in 2019 and 2020 [22,23]. It included data for general characteristics, including socio-demographic characteristics, anthropometric measurements, lifestyle characteristics, the 24-h Dietary Recall, Food Frequency Questionnaires (FFQ-21), and the questionnaire to determine the dietary and the physical activity habits of individuals, which was presented to the participants in a written form. The survey was performed as face-to-face interviews with the participants. It was self-administered, but help from the attending dietitian was available, if requested. Understanding of the questionnaire and survey were thus supported by the participating expert.

The 24-h Dietary Recall and FFQ-21 were used for detailed determination of all amounts of food and beverages consumed by the participants [24]. They are common methods for measuring dietary patterns in large epidemiological diet and health studies. Based on the 24-h Dietary Recall, nutritional parameters, including macro- and micronutrients, were assessed. Intake of energy, carbohydrate, protein, lipids (cholesterol, saturated-, monounsaturated-, and polyunsaturated fatty acids), dietary fiber, vitamins (retinol, carotene, A, D, E, K, B1, B2, B3, B5, B6, B7, B9, B12, and C), and minerals (Na, Ca, K, Cu, Fe, Mg, Zn, and P) were recorded. The FFQ-21 helped to determine the frequency of food consumption and food groups by day, week, or month [25]. For each food group (milk–yogurt, cheese, red meat, white meat, egg, legumes, fresh fruits, fresh vegetables, grains, liquid oil, and solid fat), the frequency of their intake was recorded as: “I do not eat that food”, “everyday”, “1–2 times per week”, “3–4 times per week”, “5–6 times per week”, “once per 15 day/twice per month”, or “once per month”.

Some lifestyle characteristics, such as smoking status (non-smoker/past smoker/current smoker, including frequency) alcohol consumption (non-consumer/past consumer/current consumer, including frequency), and obesity were assessed. 

Anthropometric measurements were used for calculation by weight (kg)/height (m^2^) and participants’ Body Mass Index (BMI) values, which were classified according to the WHO’s BMI standards (kg/m^2^) [26]. BMI below 18.5 was classified as underweight and BMI 18.5–24.9 as normal weight. BMI 25.0–29.9 was classified as overweight (pre-obese), 30.0–34.9 as Obesity Class I, 35.0–39.9 as Obesity Class II, and above 40 as Obesity Class III. 

Dietary habits of study participants were inquired as meal numbers per day, skipping meals, reasons for skipping meals, kind of foods consumed at snack time, frequency of eating out, frequency of fast-food consumption, the amount for daily water consumption, the most preferred taste, the presence of adding salt without tasting food, the three most preferred cooking methods. We also asked about the three most preferred foods. Physical activity habits were determined according to questions on the absence or presence of regular physical activity, type, frequency, and duration of the activity [18,19,20,22,23]. In this context, they were asked whether they regularly perform physical activity, and the questionnaire included choices about what kind of activity can be performed regularly, in various categories such as walking, running (outdoors), aerobic/step, and activities with tools such as treadmills, bicycles, swimming, and other (specify). When asked about the frequency of the physical activity, choices were presented as 1 day per week, 2 days per week, 3 days per week, 4 days per week, 5 days per week, 6 days per week, and every day. The duration of activity was asked about, and the choices were presented as less than 30 min, 30 min, 45 min, 1 h, or more than 3 h. 

### 2.3. Data Analyses

Data obtained from the 24-h Dietary Recall and the FFQ-21 tests were entered and analyzed with the BeBIS Nutrition Data System for Research software version 7.2 (Pacific Company, Istanbul, Turkey). Descriptive statistics were performed to categorize and calculate the distribution and frequency of variables (including socio-demographic data, lifestyle characteristics, BMI, dietary intake, and dietary and physical activity habits). Numerical variables were presented as means with SDs (mean ± SD) and median with minimum and maximum values. Categorical variables were expressed as number and percentage. Statistical analyses were performed using nonparametric tests, the χ2 test, and the Kolmogorov–Smirnov test for analyzing the normality, as well as the 1-sample t test, the χ2 chi-square test, and Fisher’s exact test for performing comparisons of data from the study groups. *p* < 0.05 was considered to be statistically significant. Data were analyzed using the Statistical Package for the Social Sciences version 21.0.

## 3. Results

### 3.1. General Characteristics

Based on the study of 300 individuals, 150 were diagnosed with dizziness/vertigo symptoms and 150 did not show any symptoms (non-vertigo/non-dizziness). General characteristics of the respective groups, including socio-demographic data, are listed in Table 1. The age of participants ranged between 18 and 55, and the median age was 26.5 years for the control group and 40 years for the patient group, respectively. 

The study was conducted with 241 females (80.3%) and 59 males (19.7%), while 86.0% of the control group and 74.7% of the patient group were females. Gender ratio (male/female) was 0.16:1 in the control and 0.33:1 in the patient groups. 

Age and gender were significantly associated with the patient on univariate analysis (*p* = 0.001 and *p* = 0.014, respectively). In the patient group, 83.3% (*n* = 125) were married, 78% (*n* = 117) had basic education, 52.7% (*n* = 79) were housewives, and 99.3% (*n* = 149) lived in a metropolitan area. In the control group, 60.8% (*n* = 98) and, in the patient group, 44% (*n* = 66) of individuals were employed. Significant differences were determined for marital status, working status, and education level between both groups (*p* = 0.001).

### 3.2. Lifestyle Characteristics

No differences in lifestyle factors, such as smoking habits and alcohol consumption, were observed between groups (Table 2). Also, the history of smoking/alcohol and current smoking/alcohol habits did not differ significantly between both groups (*p =* 0.810, 0.700, 0.127, and 0.060, respectively). A total of 6% (*n =* 9) of the control group and 7.3% (*n =* 11) in the patient group defined their smoking frequency as “sometimes”, while 37 (24.7%) participants in the control and 23 (15.3%) in the patient group stated to smoke “always”. Alcohol consumption was defined as “always” by 17 (11.3%) individuals in the control and 8 (5.35%) participants in the patient groups. 

The obesity status obtained according to BMI calculation from anthropometric measurements revealed that only a third (34.7%, *n =* 52) of the patient group but more than half (54%, *n =* 81) of the control group were underweight (2% versus 10.6%) or of normal weight (32.7% versus 43.3%), whereas 65% of the patient group (*n =* 98) were overweight or obese. These differences were significant (*p =* 0.001) (Table 2).

### 3.3. Dietary Habits

When asked how many meals the study participants ate per day, no significant difference was found between groups (*p =* 0.246). A significant difference was seen for skipping meals (*p =* 0.044), and the rate was 36% (*n =* 54) for meal skipping “everyday” in the patient group. Participants in the control group skipped meals “everyday” at a rate of 28.7% (*n =* 43) and “sometimes” 56% (*n =* 84), while 40.6% (*n =* 61) of the patient group answered “sometimes”. In total, 127 adults in the control group and 115 adults in the patient group skipped meals. Lunch was the most skipped meal for nearly half of both groups, followed by breakfast and dinner (*p =* 0.123). The main reason for skipping meals was lack of time in 41.3% in the control and 42.7% in the patient groups (*p =* 0.300). 

When asked if snacks were consumed, 89.3% (*n =* 134) of the control and 86% (*n =* 129) of the patient groups declared “yes” (*p =* 0.194). The most consumed snack was fruits (*n =* 76 and *n =* 79) in the control (50.7%) and the patient (52.7%) groups (*p =* 0.729). Dairy products (*n =* 45; 30%), biscuits (*n =* 41; 27.3%), nuts (*n =* 37; 24.7%), and chocolate (*n =* 24; 16%) follow fruits as snacks in the patient group. Following fruits, consumption of chocolate and nuts were found at the same rate in the control group (*n =* 35; 23.3%). Biscuits (*n =* 29; 19.3%) and dairy products (*n =* 25; 16.6%) were also consumed by the control group. Significant differences were determined for the dairy products and biscuits as the preferred snack among both groups (*p =* 0.001), in favor of the patient group. Bakery products and chips were preferred at less than 10% in this study.

A total of 11%(*n =* 16) of the control and 25% (*n =* 38) of the patient groups reported their frequency of eating out as “never”, whereas nearly half (*n =* 71) of the control group and 30% (*n =* 45) of the patient group indicated that they eat out once a week or more often. A total of 18% (*n =* 27) of the control group and 24% (*n =* 36) of the patient group were eating out once per month. Differences between both groups were statistically significant (*p =* 0.008). When asked about fast-food consumption, it was found that 42% of the control group and 69% of the patient group (*n =* 63 and 103, respectively) “never” consumed fast-food, whereas 35% and 21% (*n =* 53 and 31, respectively) did once per month (*p =* 0.001). 

The most preferred tastes of food were sweet in the patient group and salty in the control group. No significant difference was observed between the groups (*p* = 0.137). Furthermore, 24% of the control (*n =* 36) and 19% of the patient groups (*n =* 29) added salt to food without tasting first (*p =* 0.295). 

The three most preferred cooking methods in the control group were oven baking (*n* = 113; 75%), boiling (*n =* 108; 72%), and frying (*n =* 90; 60%), whereas the results in the patient group were boiling (*n =* 123; 82%), oven baking (*n =* 113; 75%), and frying (*n =* 82; 55%). There was a statistically significant difference for boiling between the groups (*p =* 0.04), in favor of the patient group. 

Participants were also asked about their three most preferred foods. The USDA food groups were defined as fruits and vegetables, grains, protein foods, and dairy [27]. In this study, all food groups were inquired with explanation (red meat, red meat products, chicken meat, fish, milk, milk products, fruits, vegetables, grains, bakery, and sweet foods). Over half of the participants in this study ranked chicken meat as the first choice of food (*p =* 0.908). The three most preferred foods in the control group were chicken meat (*n =* 80; 53%), red meat (*n =* 76; 50%), and milk products (*n =* 74; 49%), while the ranking in the patient group was chicken meat (*n =* 81; 54%), milk products (*n =* 80; 53%), and red meat (*n =* 69, 46%). There was no significant difference for these foods between groups (*p =* 0.908, 0.419, and 0.488, respectively). Fish was the ninth most frequently consumed food in the control (*n =* 19; 13%) group, but it was seventh in the patient group (*n =* 34; 23%). For fish consumption, there was a significant difference between the groups (*p =* 0.023), in favor of the patient group. The consumption rate of fresh fruit and vegetables was at the same low level (*n =* 68; 45%) in both groups. 

The daily water consumption of both groups was between 1 and 5 L. It was found that the patient group consumed statistically significant lower amounts of water per day than the control group (*p =* 0.026).

### 3.4. Nutritional Assessment

The nutritional assessments were performed according to 24-h Dietary Recall and the FFQ-21 questionnaires, and the obtained data of both groups were recorded and statistically compared. The frequency of consumed food groups was determined according to the FFQ-21 test. 

It was found to be statistically significant that the patient group (*n =* 142; 94.7%) consumed “every day” more bread than the control group (*n =* 120; 80%) (*p =* 0.001). The general frequency of grain consumption was statistically significant between the groups (*p =* 0.05), in favor of the patient group. 

Dairy products were more often consumed daily by the patient group (*n =* 121) than the control group (*n =* 94) (*p =* 0.006), but the consumption of milk was insignificant in comparison to the control group (*p =* 0.345). 

Daily consumption of bread, milk, dairy products, eggs, fruits, and vegetables showed a high rate for every day without insignificant differences. The consumption rates of red meat “1 or 2 times per week”, white meat “2 or 3 times per week”, and legumes “everyday” were comparable in both groups (*p* = 0.468, 0.529, and 0.315, respectively). In both groups, the rate of eating legumes and grains only “1–2 times/week” was higher than for “everyday” intake. No significant differences between the groups were determined for liquid oils and solid fats. 

Nutritional parameters, including nutrients gained from the 24-h Dietary Recall records, are given in Table 3, which shows the energy and nutrient intakes of the study participants for 24 h. No significant differences for energy intake were found between the groups (*p* = 0.656). Intake of carbohydrates, fats, and proteins in grams, as well as in percentages, and dietary fiber intake in grams varied insignificantly between the groups. Only the intake of carotene in milligrams, and vitamins D and K in micrograms, showed statistically significant differences between the groups (*p* < 0.05). The levels of dietary intake for carotene and vitamin K were significantly lower in the patient group than the control group, but the intake of vitamin D was the opposite of these.

### 3.5. Physical Activity Habits

Physical activity was defined as any body movement produced by muscle contraction that results in higher energy expenditure compared to basal resting levels [28]. The World Health Organization has recommended regular physical activity as an effective non-pharmacological method that contributes to the quality of life of individuals [26]. Therefore, the physical activity habits of participants were determined, specifically the absence or presence of regular physical activity, along with the type, frequency, and duration of the activity. Physical activity of participants was assessed as hours per day and per week and classified with intensity indication (moderate, vigorous, etc.).

Differences in physical activity habits were not statistically significant between both groups (*p =* 0.172). The frequency of regular “everyday” physical activity was at a very low level in the control group (*n =* 27; 18%) and was even lower in the patient group (*n =* 14; 9%) (*p =* 0.488). The frequency for regular physical activity “five-times per week” was almost equally low in the control (*n =* 9; 6%) and the patient (*n =* 8; 5%) groups.

Also, the duration of daily regular physical activity was similarly low in both groups. “30 min” of activity were recorded as 12% in the control (*n =* 18) group and 8% in the patient (*n =* 12) group, while “1 h” was 7% in the control (*n =* 11) group and 9% in the patient (*n =* 14) group (*p =* 0.505). The most preferred type of regular physical activity in both groups was walking “a moderate- physical activity”, for which the difference was statistically significant in favor of the control group (*p =* 0.037). Performing vigorous-physical activity in both groups was low (7% and 9%, respectively).

In the control group, six participants (4%) walked daily for 3.5 h per week, and eight participants (5%) walked for 5.25 h per week. Nine individuals (6%) walked between 7 and 21 h per week. In the patient group, four participants (3%) regularly walked for 3.5 h per week, and three participants walked for 5.25 h per week, whereas four individuals (3%) walked between 7 and 21 h per week. On the other hand, performing physical activity varied between 30 min and 3 h per day in both groups, and only one participant in the control group walked less than 30 min per day weekly. 

As a result, 35% (*n* = 53) of the control group performed regular physical activity, and 29% (*n* = 43) of them preferred “walking”. Only 23 (15%) of them walk “≥30 min everyday”. The result was ≥210 min per week in the control group. On the other hand, 28% (*n* = 42) of the patient group performed physical activity regularly, and 19% (*n* = 28) of them chose “walking”. Eleven individuals (7%) walked “≥30 min everyday” in the patient group. 

## 4. Discussion

Dizziness (including vertigo) affects about 15% to over 30% of adults yearly, according to large population-based studies [1,4]. Although dizziness is a common symptom in both primary care and referral settings, the relative frequency of the various causes is not well defined [29]. Therefore, nutritional assessment, including dietary and physical activity habits, in adults with and without dizziness/vertigo were investigated in this comparative study, which provides detailed information about dietary patterns of study participants. 

Vertigo is the most common type of dizziness and BPPV in adults [1], and its prevalence is higher in women than in men, with a 2003 survey showing that the prevalence of BPPV was 3.2% in females, 1.6% in males, and 2.4% overall [30]. The current study supports the previous research on gender differences with a statistically significant result. In addition, our comparison of marital status, education, and work statutes showed significant differences between the control and patient groups. 

A few cross-sectional studies conducted in Brazil included individuals aged 60 years and older [16,19]. Recently, a Korean study was conducted with individuals aged 40 years and over, where a significant difference was found between age groups among MD and control groups [13]. Similarly, participants in our study were 18–55 years old, with a mean age of 40.5 ± 9.4 years in the patient group.

Lifestyle modification can be a first approach for the management of dizziness/vertigo [31], as smoking status, alcohol consumption, and obesity are non-invasive, gradually changeable, and cost-effective factors [32]. On the other hand, the global trend regarding unhealthy lifestyle behaviors parallels the worldwide increase in chronic diseases [33]. Some studies have reported the effects of nutrition or nutritional modifications on MD or BPPV patients [10,16]. However, there was no consistent evidence for the impact of these lifestyle factors and no supporting evidence for the association between being underweight and the occurrence of vertigo. A few reports suggested the risk of vertigo in patients with obesity [13,34,35]. On the other hand, a study in Turkey reported that dizziness was more common in smokers than non-smokers and ex-smokers [4]. Another study suggested that smoking may increase the risk of MD, while alcohol consumption may decrease the risk of MD [13]. In the present study, no differences were observed regarding the smoking status or alcohol consumption of participants with or without dizziness/vertigo. However, the obesity rate was statistically significantly higher in the patient group compared to the control group. Similarly, a study from the UK reported that the obesity rate was higher in MD patients than in control individuals [14]. Additionally, being underweight was negatively associated with MD in this cohort study.

Some dietary habits are considered to be risk factors for various changes in the human body, while changes in diet can reduce the symptoms of disease and improve quality of life [36]. Low sodium diets, reduced daily caffeine and alcohol consumption, a gluten-free diet, and consumption of specially proceeded grains may help to keep MD attacks under control [10]. In the current study, dietary habits were inquired using a questionnaire [22,23] under the supervision of a specialist. Significant differences were found for skipped meals, eating out once per month, and daily water consumption. The regulation of water intake and hydration is required for homeostasis in the human body [37]. Daily water consumption in the patient group was lower than in the control group in the present study. This finding may suggest the loss of homeostasis and an imbalance of the vestibular system in these patients. A study performed with MD patients demonstrated that water intake treatment affected patients positively in correcting and preventing hearing loss [38]. The relationship between skipping meals and health and diseases has been the focus of recent research [39,40]. On the other hand, the 2020–2025 Dietary Guidelines for Americans did not include meal frequency, skipping, and intervals, with the addition “was unable to find sufficient evidence on which to summarize the evidence between frequency of eating and health” [41]. In the current study, meal skipping “everyday” was significantly high, and lunch was the most skipped meal in the patient group. These results draw attention, since a prospective study conducted in the US including adults aged > 40 years reported that skipping lunch or dinner was associated with higher risk of all-cause mortality [40]. For a healthy lifestyle, dietary guidelines recommend salt reduction in the diet [41,42], with a maximum salt intake of 5 g per day [42]. High salt consumption is one of the important factors for MD attacks [10], while a reduced salt diet provided beneficial treatment of MD patients [8,43,44]. In our study, the most preferred taste of food was salty in individuals in the patient group, of which 19% added salt to food without tasting first. The three most frequently used methods for food preparation were oven baking, boiling, and frying in both groups, but there was a statistically significant difference concerning the boiling method. Although fresh fruits and vegetables were seen as a source of dietary fiber, less than half of the participants reported their consumption at the same low level in both groups. Previously, a study reported decreased dietary fiber intakes in BPPV patients [16]. There was no significant difference for the three most frequently consumed foods between the groups investigated in our study. Fish was the ninth most frequently consumed food in the control group, but it was seventh in the patient group, and this difference was significant between the groups.

Nutritional intake and metabolism balance is required for normal physiological functioning of the human body [45]. An imbalance caused by nutritional deficiencies or excess can cause or support diseases. To the authors knowledge, this is the first study investigating energy and nutritional intake (including frequency) of dizziness/vertigo patients in comparison to a control group by means of the 24-h Dietary Recall and FFQ-21 questionnaires. In the present study, the frequency rate for the consumption of bread, milk, dairy products, eggs, fruits, and vegetables indicated as “everyday” by the participants was high in comparison to other intervals. Also, a high rate of participants rated the consumption of red meat as “1 or 2 times/week” and white meat as “2 or 3 times/week”. These results overlapped with the WHO recommendations for a healthy diet in adults [42]. However, most of the participants explained the intake of legumes and grains as “1–2 times/week”, whereas a daily intake is recommended by WHO [42]. Nutritional assessment by 24-h Dietary Recall recording showed no significant differences regarding intake of energy, carbohydrates, fats, proteins, dietary fiber, and minerals. Likewise, dietary fiber intake of BPPV patients was not significantly decreased in comparison to non-vertigo individuals in Korea [17]. Contrary to these, a cross-sectional study in Brazil found an association between BPPV and inadequate diet, leading to increased carbohydrate and polyunsaturated fatty acids intake and decreased dietary fiber intake [16]. Studies revealed that decreased serum vitamin D level may be a risk factor of BPPV [15]. Furthermore, an intake of vitamin D and calcium carbonate for 1 year resulted in the recurrences of vertigo in 24% of BPPV patients [9]. In a study determining serum vitamin D levels in positional vertigo and non-positional vertigo adults, no statistically significant differences in the vitamin D status could be observed [17]. Although serum vitamin D levels were not investigated in the present study, an intake of vitamin D based on diet was found to be similarly low in both groups, with an even lower amount in the control group. Results of the present study were supported partially by other studies [9,15,20]. Of course, measurement of serum vitamin D is a more reliable method to determine vitamin D supply in humans than the dietary and supplemental assessments [46]. Additionally, serum vitamin D levels also depend on sunlight exposure, which is a major contributor to vitamin D levels in humans. In the present study, the intakes for carotene and vitamin K were lower in the patient group than the control group, and the differences were statistically significant. Likewise, a study reported that individuals aged >40 years with vertigo showed a significantly lower intake of carotene [17]. In our study, the intake of carotene levels was very low in both groups. 

Daily physical activity is essential for a healthy life and improves the quality of life of individuals [26]. The presence of vertigo was lower in elderly patients who had regular physical activity [18]. At the same time, there was a relationship between lack of regular physical activity and vertigo. In the present study, the habit to practice 30 min of regular physical activity “every day” was, overall, very low, and it was even lower in the patient group than the control group. Based on the Physical Activity Guidelines for Americans, adults are recommended being physically active weekly, with at least 150–300 min of moderate-intensity or 75–150 min of vigorous-intensity activity [47]. In the present study, 15% of the control and only 7% of the patient groups perform physical activity in the moderate-intensity of ≥210 min per week, and 7% and 9%, respectively, performed vigorous-intensity activity. Only a few participants in each group followed the general recommendation to be physically active for 60 min per day [48]. The low degree of physical activity recorded is a concern, since it has been reported that regular physical activity decreases the risk of vertigo in women [19]. Of course, it has to be considered that significantly higher activity limitations were found in vertigo patients, who have a higher risk to fall [17], which may discourage them from being more active. Hence, professional monitoring and supervising is important to improve the patient’s quality of life and, in addition, help reduce the economic impact of vertigo and dizziness on the health system.

The limitations of this study include the lack of follow-up interviews, the possibility of pleasing responses (as opposed to completely anonymous interrogation or objective measures), and incorrect responses for some habits (lifestyle, diet, and physical activity), as the method of self-reported habits has been found to be less reliable in previous studies [22,23]. In addition, the disproportion between the groups regarding personal characteristics are another limitation of the study.

## 5. Conclusions

This work is the first study that combines the 24-h Dietary Recall and FFQ-21 questionnaire studies for nutritional assessment in dizziness/vertigo and non-vertigo/non-dizziness adults. Additionally, socio-demographic characteristics, lifestyle characteristics, dietary habits, and physical activity habits, including the absence or presence of regular physical activity, were determined and compared between both study groups. Our study confirms that female gender and increasing age are associated with dizziness/vertigo. A significantly higher obesity rate was observed in the patient group of the study, while participants of this group also skipped meals more often and had reduced water intake compared to the control group. Additionally, nutrient intake (e.g., carotene, vitamins D and K) and the consumption frequency of some food groups differed considerably. Moreover, the results revealed a notably lower degree of physical activity of participants suffering from dizziness/vertigo. All of these factors might influence dizziness/vertigo, and patients need to be educated or counseled to change their habits. Since our study focused on dizziness/vertigo in general, regardless of the underlying pathology, further studies are encouraged focusing on the nutritional status, general diet, lifestyle factors, and physical activity of patients with accurately diagnosed diseases (e.g., Meniere’s, BPPV, vestibular migraine). Our general results on dizziness/vertigo show the need for professional counseling of patients suffering from symptoms to encourage, supervise, and monitor beneficial changes in diet, lifestyle and physical activity. 

## Figures and Tables

**Table 1:** General characteristics, including socio-demographic data, of study participants. The results are given as mean ± standard deviation. * *p <* 0.05.

Characteristics	Control Group (*n =* 150)	Patient Group (*n =* 150)	*p* Value
Age (years)	31.6 ± 11.1	40.5 ± 9.4	0.001 *
Gender ratio (male/female)	0.16:1	0.33:1	0.014 *
Female	129 (86.0%)	112 (74.7%)	
Male	21 (14.0%)	38 (25.3%)	
Marital status			0.001 *
Married	82 (54.7%)	125 (83.3%)	
Single	68 (45.3%)	25 (16.7%)	
Education			0.001 *
Basic	71 (47.3%)	117 (78.0%)	
High school	79 (52.7%)	33 (22.0%)	
Work status			
Housewife	49 (37.2%)	79 (52.7%)	0.001 *
Working	98 (60.8%)	66 (44.0%)	0.001 *
Retired	3 (2.0%)	5 (3.3%)	0.642
Living in metropolitan	148 (98.7%)	149 (99.3%)	0.498

**Table 2:** Some lifestyle characteristics of study participants. The results are given as mean ± standard deviation. * *p <* 0.05. ^a^ Bonferroni corrected pairwise comparisons were conducted.

Some Lifestyle Characteristics	Control Group (*n =* 150)	Patient Group (*n =* 150)	*p* Value
Smoking status			
Non-smoker	104 (69.3%)	106 (70.6%)	0.127
Past smoker	56 (37.3%)	53 (35.3%)	0.810
Current smoker	46 (40.6%)	44 (22.6%)	0.127
Alcohol consumption			
Non-consumer	132 (88.7%)	142 (94.7%)	0.060
Past consumer	16 (10.7%)	14 (9.3%)	0.700
Current consumer	17 (11.3%)	8 (5.3%)	0.060
^a^ Obesity (BMI, kg/cm^2^)	25.2 ± 5.4	27.4 ± 5.4	0.001 *
<18.5 (Underweight)	16 (10.6%)	3 (2.0%)	
≥18.5–24.9 (Normal)	65 (43.3%)	49 (32.7%)	
≥25–29.9 (Overweight/Pre-obese)	43 (28.7%)	53 (35.3%)	
≥30–34.9 (Obesity class I)	19 (12.7%)	33 (22.0%)	
≥35–39.9 (Obesity class II)	7 (4.7%)	7 (4.7%)	
≥40 (Obesity class III)	0 (0.0%)	5 (3.3%)	

**Table 3:** Energy and nutritional intakes, including nutrients, of study participants, obtained from the 24-h Dietary Recall. The results are given as median (minimum–maximum), as well as mean ± standard deviation (S.D.). * *p <* 0.05.

Intakes	Control Group (*n =* 150)		Patient Group (*n =* 150)		*p* Value
	Median (min.–max.)	Mean ± S.D.	Median (min.–max.)	Mean ± S.D.	
Energy (kcal)	1213.78 (229.42–4210.06)	1289.21 ± 602.05	1226.89 (118.42–3139.64)	1330.85 ± 602.72	0.656
Carbohydrate (g)	120.02 (7.28–350.78)	135.30 ± 71.85	130.15 (3.67–507.17)	140.09 ± 82.68	0.889
Carbohydrate (%)	44.00 (10–69)	42.38 ± 11.64	42.00 (7.0–83.0)	41.83 ± 13.33	0.380
Fat (g)	55.67 (7.730–324.09)	59.91 ± 36.23	55.13 (1.67–172.55)	61.23 ± 29.65	0.440
Fat (%)	41.50 (16–77)	41.39 ± 10.54	43.00 (6.0–77)	42.26 ± 12.08	0.229
Protein (g)	46.57 (7.23–184.38)	49.75 ± 25.56	45.64 (3.57–139.69)	50.34 ± 29.74	0.972
Protein (%)	16.00 (6.0–39.0)	16.28 ± 5.03	15.00 (5.0–32.0)	15.47 ± 4.85	0.078
Dietary fiber (g)	13.64 (0.36–77.18)	15.01 ± 8.32	13.32 (1.54–43.12)	14.68 ± 7.34	0.685
Water intake (mL)	820.64 (124.05–2674.28)	881.47 ± 440.25	827.91 (15.44–2588.08)	894.90 ± 404.26	0.690
VITAMINS					
Vitamin A (µg)	771.65 (7.68–5932.22)	892.30 ± 678.64	738.27 (1.80–27,863.0	1145.46 ± 2465.35	0.956
Carotene (mg)	1.95 (0.00–12.33)	2.49 ± 1.95	1.56 (0.00–16.840)	2.08 ± 1.98	0.015 *
Retinol (µg)	328.90 (0.00–1396.0)	352.03 ± 222.14	330.20 (1.35–27,456.0)	653.46 ± 2390.03	0.445
Vitamin B1 (mg)	0.54 (0.08–5.82)	0.59 ± 0.49	0.53 (0.05–1.53)	0.58 ± 0.29	0.938
Vitamin B2 (mg)	0.88 (0.13–2.46)	0.94 ± 0.44	0.84 (0.02–5.68)	0.99 ± 0.67	0.674
Vitamin B3 (mg)	7.27 (1.55–93.71)	8.47 ± 8.28	7.16 (0.43–38.49)	8.48 ± 5.86	0.946
Vitamin B5 (mg)	2.89 (0.50–18.24)	3.23 ± 1.88	2.92 (0.09–12.40)	3.27 ± 1.88	0.831
Vitamin B6 (mg)	0.85 (0.19–3.12)	0.91 ± 0.42	0.81 (0.04–2.01)	0.88 ± 0.40	0.595
Vitamin B7 (µg)	25.16 (1.20–223.92)	27.07 ± 20.73	26.48 (1.35–163.54)	29.46 ± 20.72	0.392
Vitamin B9 (µg)	211.86 (25.25–1153.50)	223.27 ± 123.25	193.30 (4.95–538.0)	220.58 ± 105.15	0.746
Vitamin B12 (µg)	2.00 (0.00–10.73)	2.49 ± 2.05	2.10 (0.00–101.70)	3.51 ± 8.85	0.771
Vitamin C (mg)	59.22 (0.00–301.20)	71.59 ± 54.98	51.64 (0.00–257.72)	64.86 ± 53.23	0.182
Vitamin D (µg)	1.02 (0.00–6.35)	1.13 ± 1.06	1.45 (0.00–13.83)	1.55 ± 1.78	0.045 *
Vitamin E (mg)	8.25 (1.30–61.30)	12.08 ± 9.48	8.38 (0.12–33.15)	10.67 ± 6.54	0.329
Vitamin K (µg)	234.50 (2.25–728.45)	259.20 ± 151.26	194.52 (4.05–549.20)	208.92 ± 128.85	0.007 *
MINERALS					
Sodium (mg)	2604.29 (215.16–53,156.92)	3127.85 ± 4444.07	2437.36 (193.95–8002.93)	2661.09 ± 1270.59	0.751
Potassium (mg)	1515.84 (197.55–5465.70)	1615.48 ± 771.36	1518.97 (53.1–3797.58)	1566.48 ± 702.58	0.643
Calcium (mg)	467.55 (29.20–1505.25)	502.87 ± 272.38	414.35 (22.50–1327.84)	468.25 ± 257.29	0.186
Magnesium (mg)	165.99 (26.15–1106.10)	182.61 ± 106.02	167.61 (16.65–612.98)	185.47 ± 93.92	0.875
Phosphorus (mg)	757.79 (149.56–2810.0)	793.81 ± 383.72	731.30 (46.35–2037.52)	798.62 ± 394.80	0.968
Iron (mg)	7.75 (1.25–17.74)	7.80 ± 3.21	6.96 (0.78–22.98)	7.82 ± 3.82	0.624
Zinc (mg)	6.34 (1.16–21.19)	6.87 ± 3.42	6.45 (0.56–23.30)	7.16 ± 3.92	0.833
Copper (mg)	1.06 (0.19–5.15)	1.15 ± 0.59	1.08 (0.11–5.06)	1.18 ± 0.65	0.998
LIPIDS					
Cholesterol (mg)	219.30 (0.00–885.05)	232.99 ± 162.67	229.0 (0.00–1219.10)	271.47 ± 207.65	0.190
Saturated FA (g)	19.86 (2.16–75.69)	22.63 ± 12.86	20.72 (0.29–76.51)	23.71 ± 13.34	0.570
Polyunsaturated FA (g)	9.68 (1.54–85.08)	12.13 ± 10.39	10.17 (0.69–41.50)	11.84 ± 8.23	0.959
Monounsaturated FA (g)	19.37 (2.86–152.03)	21.21 ± 15.19	19.92 (0.54–73.08)	21.33 ± 10.69	0.362
Salt intake (g)	6.10 (0.38–129.34)	7.57 ± 10.80	5.59 (0.49–19.39)	7.57 ± 3.07	0.385

Abbreviations: FA. fatty acids.

## Data Availability

Not applicable.
